# Genome-wide association mapping of leaf mass traits in a Vietnamese rice landrace panel

**DOI:** 10.1371/journal.pone.0219274

**Published:** 2019-07-08

**Authors:** Giang Thi Hoang, Pascal Gantet, Kien Huu Nguyen, Nhung Thi Phuong Phung, Loan Thi Ha, Tuan Thanh Nguyen, Michel Lebrun, Brigitte Courtois, Xuan Hoi Pham

**Affiliations:** 1 Agricultural Genetics Institute, National Key Laboratory for Plant Cell Biotechnology, Hanoi, Vietnam; 2 University of Science and Technology of Hanoi, Vietnam; 3 IRD, Université de Montpellier, Vietnam; 4 Université de Montpellier, IRD, UMR DIADE, France; 5 Palacký University Olomouc, Centre of the Region Haná for Biotechnological and Agricultural Research, Department of Molecular Biology, Šlechtitelů 27, Czech Republic; 6 Vietnam National University of Agriculture, Faculty of Agronomy, Department of Genetics and Plant Breeding, Vietnam; 7 Université de Montpellier, IRD, UMR LSTM, France; 8 Cirad, UMR AGAP, France; 9 Université de Montpellier, CIRAD, INRA, Montpellier SupAgro, Montpellier, France; Osmania University, INDIA

## Abstract

Leaf traits are often strongly correlated with yield, which poses a major challenge in rice breeding. In the present study, using a panel of Vietnamese rice landraces genotyped with 21,623 single-nucleotide polymorphism markers, a genome-wide association study (GWAS) was conducted for several leaf traits during the vegetative stage. Vietnamese landraces are often poorly represented in panels used for GWAS, even though they are adapted to contrasting agrosystems and can contain original, valuable genetic determinants. A panel of 180 rice varieties was grown in pots for four weeks with three replicates under nethouse conditions. Different leaf traits were measured on the second fully expanded leaf of the main tiller, which often plays a major role in determining the photosynthetic capacity of the plant. The leaf fresh weight, turgid weight and dry weight were measured; then, from these measurements, the relative tissue weight and leaf dry matter percentage were computed. The leaf dry matter percentage can be considered a proxy for the photosynthetic efficiency per unit leaf area, which contributes to yield. By a GWAS, thirteen QTLs associated with these leaf traits were identified. Eleven QTLs were identified for fresh weight, eleven for turgid weight, one for dry weight, one for relative tissue weight and one for leaf dry matter percentage. Eleven QTLs presented associations with several traits, suggesting that these traits share common genetic determinants, while one QTL was specific to leaf dry matter percentage and one QTL was specific to relative tissue weight. Interestingly, some of these QTLs colocalize with leaf- or yield-related QTLs previously identified using other material. Several genes within these QTLs with a known function in leaf development or physiology are reviewed.

## Background

The leaf is the primary organ used for photosynthesis and transpiration and affects yield performance in crops [1]. Leaf traits (size, shape, number, and angle) are major determinants of plant architecture and photosynthetic potential and play critical roles in determining plant yield [2, 3]. As a result, improving leaf traits can increase grain yield in ideotype breeding [4, 5]. Several previous studies have indicated that over 80% of the total assimilates in rice grains are generated from the top two leaves [6, 7], while more than 50% are produced by the flag leaf [7]. Other researchers recently confirmed the large contribution of the top three leaves of a rice plant to high photosynthetic efficiency and grain yield [8, 9]. Because the flag leaf is the main source of photosynthetic products supplied to the panicle, its removal significantly reduces 1,000-grain weight and panicle yield [10, 11, 12, 13, 14].

In rice, among leaf traits, leaf size and shape have been the most studied. Both leaf length and leaf width are strongly related to leaf area [5]. However, leaf length negatively affects leaf angle, i.e., plants with long leaves generally have a wide leaf angle due to leaf drooping. Conversely, the small leaf angle of an erect plant is associated with short and narrow leaves, which can lead to increased light capture, ultimately enhancing photosynthetic activity and yielding capacity [15]. Therefore, short and narrow leaves are more desirable than longer and wider ones. Leaf thickness is also an important leaf trait [16]. An increase in leaf thickness correlates with a decrease in leaf angle. In addition, thick leaves usually have a high density of chlorophyll and consequently a high photosynthetic efficiency per unit leaf area [17, 18]. Therefore, leaf thickness correlates positively with grain yield [16, 19]. Despite this positive correlation, leaf thickness is difficult and time consuming to measure and is usually not directly monitored in breeding programs. Leaf thickness can be approximated with other parameters, such as specific leaf weight (mass per area), specific leaf area (area per mass) and leaf dry matter percentage (also called the dry mass to fresh mass ratio) [20, 21, 22].

Compared with the size and shape of leaves, there are very few studies on leaf mass in rice [22]. However, leaf fresh weight and leaf dry weight are important growth parameters that are directly associated with water content and efficient accumulation of dry matter in leaves. Leaf dry matter percentage contributes to plant biomass production, efficient conservation of nutrients and plant responses to environmental change [23]. Leaf dry matter percentage also explains variation in potential relative growth rate [24]. Despite the importance of these leaf mass traits, their genetic basis is still not fully understood. Only a limited number of leaf traits, such as leaf length and leaf width, have been widely studied in rice.

Most studies aiming to better understand the genetic determinants of leaf traits are based on screens of mutants [25, 26, 27] and detection of quantitative trait loci (QTLs) in mapping populations [1, 28, 29, 30, 31]. Several mutant genes related to different leaf traits have been isolated and characterized. For instance, *narrow leaf 1 (nal1*) mutant plants exhibit decreased leaf width and defective vascular system development [26]. Similarly, the *NAL2* and *NAL3* genes control leaf width and vascular patterning during leaf development [32, 33]. *nal7* mutant plants have reduced leaf width with curling [25], and mutation of *NRL1* (*NARROW AND ROLLED LEAF 1*) decreases leaf width and induces semirolled leaves [27]. In contrast to the small number of genes identified in mutant analyses, many QTLs associated with leaf size have been identified. [31] detected 43 QTLs for the length, width, and length/width ratio of the flag leaf and plant yield using recombinant inbred lines and particularly found two common QTLs for flag leaf width and plant yield. [30] identified 8 QTLs for leaf length and width in IR64-derived introgression lines. In a chromosome segment substitution line (CSSL) population, [34] found 14 QTLs for flag leaf length and 9 QTLs for flag leaf width and then confirmed the function of the candidate gene *GRAIN NUMBER*, *PLANT HEIGHT*, *AND HEADING DATE 7*.*1* (*GHD7*.*1*) in increasing flag leaf size (in length and width) by using CRISPR/CAS9 targeted mutagenesis. This confirms that flag leaf characteristics are involved in yield. A total of 52 QTLs for leaf shape were detected in a double-haploid (DH) population derived from the CJ06/TN1 cross [35]. A genome-wide association study (GWAS) recently carried out on a panel of 533 rice accessions for 29 leaf traits identified a large number of novel QTLs for leaf size, shape, and color [36]. Thus, genome mapping offers an effective approach for discovering desirable QTLs and identifying candidate genes related to leaf traits that can be utilized in breeding programs.

In this study, we conducted a GWAS of five leaf mass traits in a panel of 180 Vietnamese rice landraces originated from various agrosystems across Vietnam and composed of both indica and japonica accessions [37]. Vietnam has an extremely genetically diverse collection of traditional rice varieties, which has been understudied. This panel constitutes a relevant source of new, valuable QTLs controlling root or panicle structure or water deficit resistance [37–40]. Here, we reveal that this panel presents significant phenotypic variation in leaf-related traits and specifically detect a strong correlation among the three primary leaf mass traits. By a GWAS, several QTLs for leaf mass traits were identified in the full panel and in the indica subpopulation. The most significant QTL, mapped to chromosome 10, was associated with leaf fresh weight, leaf turgid weight, and leaf dry weight. Candidate genes underlying the major identified QTLs were researched, and their possible function in regard to the corresponding trait is discussed.

## Materials and methods

### Plant materials and genotypic data

In this study, we used a panel of 183 rice accessions, including 180 Vietnamese rice landraces and three reference varieties (Nipponbare, IR64 and Azucena), whose detailed information is provided in Table A in S1 Table. Vietnamese rice landrace accessions were collected from different geographical regions of Vietnam and are adapted to different adverse agrosystems. The accessions used in this study were collected and are available in the Plant Resources Center (PRC, Ankhanh commune, Hoaiduc district, Hanoi city, Vietnam) that acts for the Vietnamese government to collect the seeds of traditionnal varieties and so is authorized institution to do it in all Vietnamese territories. A contract between Agricultural Research Institute (AGI) and PRC, authorizes AGI to have access and to study these rice accessions. This panel represents the two major subspecies of *Oryza sativa*, including indica (113 accessions) and japonica (64 accessions), with the 6 other accessions being admixed. The panel was genotyped using the diversity array technology sequencing (DArTseq) technique [41]. This genotyping revealed 21,623 SNP markers distributed throughout the genome for the full panel, with 13,814 and 8,821 SNP markers for the indica and japonica subpopulations, respectively [37]. The called SNP markers were present in more than 80% of accessions in the panel and were polymorphic with a minor allele frequency above 5% [37].

### Phenotyping

Phenotyping of leaf mass traits was conducted in summer 2015 in a nethouse located at the Van Giang experimental station, Vietnam (20°54’8”N and 105°57’4”E). The Van Giang experimental station belongs to AGI and is dedicated for these kinds of essays. Any damage to endangered or protected species was generated. The experiment was designed with 3 replicates and 50-cm spacing between replicates. Within each replicate, the 183 rice accessions were randomly distributed in 3 adjacent rows of 61 rubber pots (25*30*40 cm). The accessions were separately sown in seedling beds for 7 days and then transplanted into the rubber pots, with 10 plants per pot. Leaf samples were collected four weeks after transplantation. A 7-cm-long leaf fragment was accurately cut from the middle of the second fully expanded leaf of the main tiller of each plant and immediately put into a small plastic ziplock bag of known weight. The bags with samples were weighed to determine the leaf fresh weight (FW, mg). Then, the samples were put into falcon tubes containing distilled water overnight and weighed again to obtain the leaf turgid weight (TW, mg). Afterwards, the samples were oven dried at 70°C for 3 days to determine the leaf dry weight (DW, mg). The relative tissue weight (RTW) was calculated as the ratio of leaf fresh weight to turgid weight. It represents an estimate of the relative water content, which is related to the water status of the plant [42]. The leaf dry matter percentage (LDMP, %) was computed as (DW/FW)*100 and is related to the dry matter production and growth rate of the plant [23, 24].

### Statistical analysis

Phenotypic data were analyzed using R software version 3.5.1 to estimate the means, standard deviations, coefficients of variation, variances and broad-sense heritabilities for each trait. The broad-sense heritability (H^**2**^) was estimated by using the genotypic and phenotypic variances as follows: H^**2**^ = (F-1)/F, where F is the F-value from ANOVA for the genotype factor. Phenotypic correlations among the traits were computed by Pearson’s method, using the R corrplot package. All statistical graphs were created using R.

### Genome-wide association analysis

An association analysis was conducted using a mixed linear model (MLM) in TASSEL v.5.2.48. The structure matrix was determined by running a PCA (principal component analysis) of the genotypic data, which established six PCA axes for the full panel and the indica subpopulation and four for the japonica subpopulation [37]. A kinship matrix was generated using the IBS (pairwise identity-by-state) method to account for the relatedness among accessions. MLM analysis was applied using the P3D (population parameters previously determined) method without compression. The SNP-trait associations were declared significant when P-value < 1e-04.

Pairwise linkage disequilibrium (LD) was calculated between significant SNPs and the surrounding SNPs using the LDheatmap R package. The QTL regions were defined in LD blocks with an r-square cutoff of 0.4. For low-LD blocks (< 50 kb), the QTL interval was expanded to a distance of +/- 50 kb. For markers isolated within an LD block to be significant, we required a minor allele frequency (MAF) above 10%.

## Results

### Phenotypic variation and heritability of the leaf mass traits

To evaluate panel variability at the phenotypic level, an ANOVA was conducted for the full panel and the indica and japonica subpopulations (Table 1). The replicate effect was not significant while the variety effect was highly significant for all of the traits except RTW. The traits with a significant variety effect exhibited high values of broad-sense heritability (H^2^), varying from 0.29 to 0.80 in the full panel but lower in the indica (0.29–0.69) and japonica (0.61–0.69) subpopulations. The coefficient of variation (CV) of the traits ranged from low to moderate (2.1–23.8%), while minor variation was observed for RTW.

**Table 1:** Phenotypic variation and trait broad-sense heritability for the three populations.

Traits	n	mean	sd	CV	Rep	Accession	F-value	H2
***Full panel***								
FW	183	68.72	16.30	23.72	0.8653	<0.001	4.96	0.80
TW	183	70.84	16.85	23.79	0.6327	<0.001	5.06	0.80
DW	183	18.18	3.28	18.04	0.7637	<0.001	3.38	0.70
RTW	183	0.97	0.02	2.06	0.0128	0.4038	1.03	0.03
LDMP	183	26.94	2.38	8.83	0.7494	0.0156	1.41	0.29
***Indica subpopulation***								
FW	113	60.9	11.37	18.67	0.8554	<0.001	2.92	0.66
TW	113	62.87	11.98	19.06	0.9320	<0.001	3.27	0.69
DW	113	16.9	2.67	15.80	0.9659	<0.001	2.55	0.61
RTW	113	0.96	0.02	2.08	0.2125	0.3828	1.05	0.05
LDMP	113	28.05	1.91	6.81	0.4227	0.0156	1.41	0.29
***Japonica subpopulation***								
FW	64	82.42	15.09	18.31	0.3746	<0.001	3.22	0.69
TW	64	84.86	15.51	18.28	0.2309	<0.001	3.06	0.67
DW	64	20.38	3.14	15.41	0.4454	<0.001	2.55	0.61
RTW	64	0.97	0.02	2.06	0.0796	0.3122	1.11	0.10
LDMP	64	25.01	1.91	7.64	0.0302	<0.001	2.88	0.65

n: number of accessions; Rep: replication; FW: leaf fresh weight; TW: leaf turgid weight; DW: leaf dry weight; RTW: relative tissue weight; LDMP: leaf dry matter percentage.

**Fig 1:**
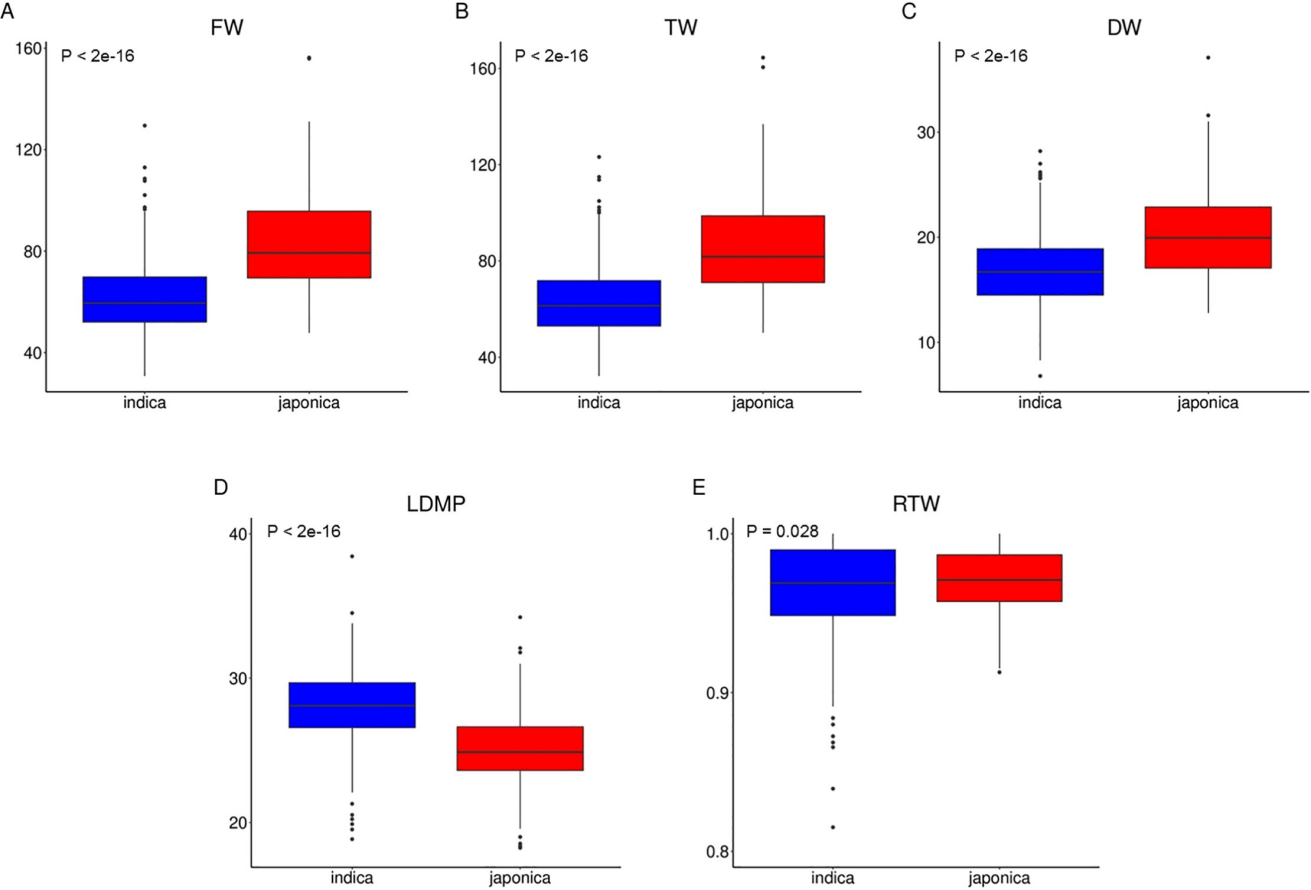
Boxplots of the distribution of leaf mass traits. Indica subpopulation in blue; japonica subpopulation in red; FW: leaf fresh weight; TW: leaf turgid weight; DW: leaf dry weight; RTW: relative tissue weight; LDMP: leaf dry matter percentage. Statistical significance (ANOVA p-values) between the two subpopulations is indicated.

The means of all traits significantly differed between the indica and japonica subpopulations: the FW, TW and DW of the japonica subpopulation were 20–35% greater than those of the indica subpopulation (Table 1, Fig 1). However, by contrast, the LDMP was 11% greater in the indica subpopulation. This indicated that japonica rice accessions have heavier leaves, larger leaves, a higher leaf water content, and more rapid leaf biomass production than the indica accessions.

The phenotypic correlations among the traits displayed similar trends within the full panel and the two subpopulations (Table 2, S1 Fig). The three directly measured traits (i.e., FW, TW, and DW) were strongly positively correlated among themselves, with correlation coefficients over 0.89 (P < 0.001). Of these three traits, FW and TW were also moderately negatively correlated with LDMP (less than -0.50 in the full panel, P < 0.001). However, DW showed a weak correlation with LDMP (-0.19 in the full panel, P < 0.001). RTW was not significantly correlated with FW, TW, or DW (S1 Fig). RTW was correlated only with LDMP, but at a low level (R = -0.21 –-0.28, P ≤ 0.001).

**Table 2:** Correlation matrix of leaf mass traits in the three populations (below the diagonal). Probabilities are displayed above the diagonal (in bold, significant at P < 0.05).

Traits		FW	TW	DW	RTW	LDMP
FW	F	**1**	**< 0.001**	**< 0.001**	0.040	**< 0.001**
FW	I	**1**	**< 0.001**	**< 0.001**	0.092	**< 0.001**
FW	J	**1**	**< 0.001**	**< 0.001**	0.659	**< 0.001**
TW	F	**0.99**	**1**	**< 0.001**	0.208	**< 0.001**
TW	I	**0.98**	**1**	**< 0.001**	0.074	**< 0.001**
TW	J	**0.99**	**1**	**< 0.001**	0.202	**< 0.001**
DW	F	**0.91**	**0.91**	**1**	0.571	**< 0.001**
DW	I	**0.89**	**0.91**	**1**	0.304	0.9844
DW	J	**0.90**	**0.90**	**1**	0.507	0.4556
RTW	F	0.09	-0.05	-0.02	**1**	**< 0.001**
RTW	I	0.09	-0.10	-0.06	**1**	**0.001**
RTW	J	0.03	-0.09	-0.05	**1**	**< 0.001**
LDMP	F	**-0.55**	**-0.51**	**-0.19**	**-0.21**	**1**
LDMP	I	**-0.40**	**-0.34**	0.00	**-0.18**	**1**
LDMP	J	**-0.47**	**-0.43**	-0.05	**-0.28**	**1**

F: full panel; I: indica subpopulation; J: japonica subpopulation; FW: leaf fresh weight; TW: leaf turgid weight; DW: leaf dry weight; RTW: relative tissue weight; LDMP: leaf dry matter percentage.

### Genome-wide association mapping

**Fig 2:**
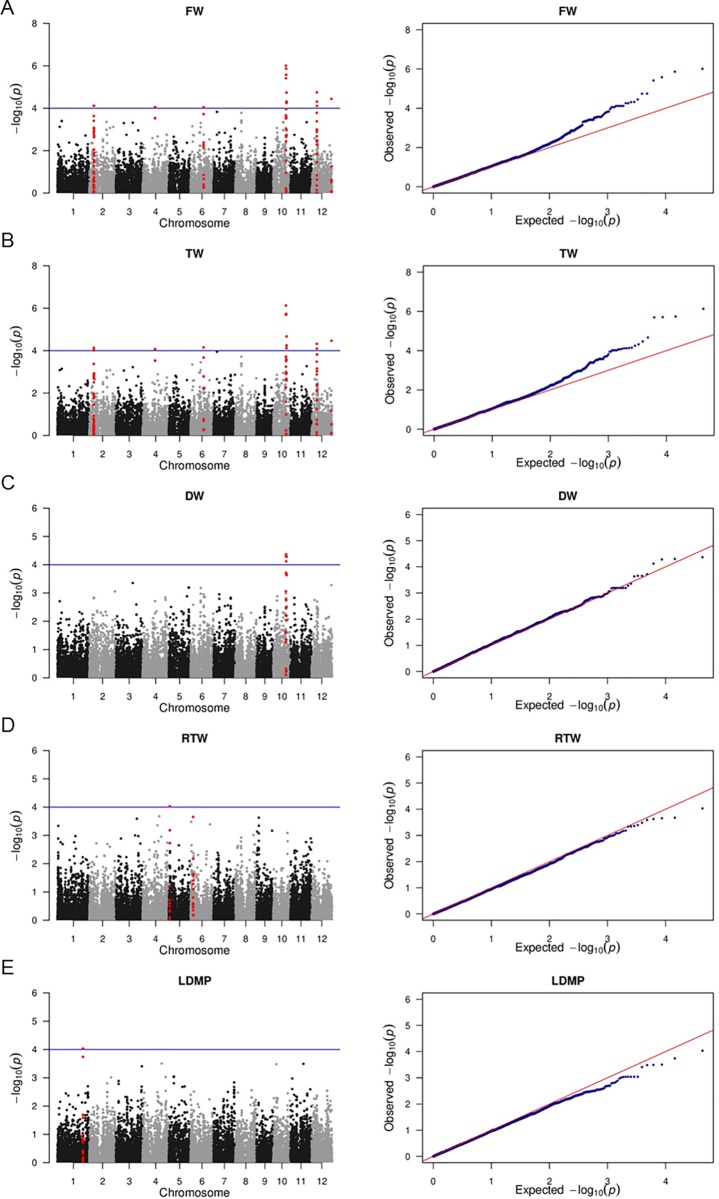
**Manhattan plots (left) and Q-Q plots (right) for the genome-wide association study of leaf mass traits in the full panel.** A: leaf fresh weight, FW; B: leaf turgid weight, TW; C: leaf dry weight, DW; C: relative tissue weight, RTW; D: leaf dry matter percentage, LDMP.

To identify association signals for the five investigated leaf mass traits, we performed a GWAS using a linear mixed model for the full panel and then independently for the indica and japonica subpopulations. As in previous studies with the same rice panel and genotypic data [38–40], the significance threshold was set at P < 1e-04. When the intervals of QTLs were determined, the threshold for significant associations underlying the detected QTLs was enlarged to P < 5e-04. As a result, a total of 103 SNP-trait associations (P < 5e-04) were detected, which consisted of 83 associations detected in the full panel and 20 in the indica subpopulation. No associations were discovered in the japonica subpopulation. In addition, the number of associations widely varied from trait to trait: 43 associations for FW as well as TW, 8 for DW, 4 for RTW, and 4 for LDMP (Table B in S1 Table). The Manhattan and QQ plots for all five traits are presented in Fig 2 for the full panel and in S2 Fig for the indica subpopulation. Since most of the significant SNP markers were common to at least two different traits or shared between the full panel and the indica subpopulation, 50 significant SNPs are listed from the total of 103 detected associations (Table B in S1 Table). Therefore, 14 QTLs were identified, whose genomic regions are highlighted in red in the Manhattan plot of each trait (Fig 2 and S2 Fig). The detected QTLs were distributed across 8 chromosomes: 1, 2, 3, 4, 5, 6, 10 and 12. Of these 14 QTLs, a QTL on chromosome 5 for RTW was generated from a single significant marker (P = 0.35e-05), which had a low minor allele frequency (MAF = 5.5%). For this reason, we removed it from further analysis. Thus, only 13 QTLs were considered significant, which were composed of 49 significant SNPs in 102 associations with different traits. Among these QTLs, six (i.e., QTL_4, QTL_5, QTL_8, QTL_11, QTL_12, and QTL_13) were specifically detected in the full panel, and four QTLs (QTL_2, QTL_3, QTL_6, and QTL_7) were specifically detected in the indica subpopulation (Table 3). QTL_1, QTL_9 and QTL_10 were detected in both the full panel and the indica subpopulation.

**Table 3:** Candidate genes underlying the identified QTLs.

QTL name	Chr	No of sig.SNPs	Traits	Population	QTL position (bp)	Gene ID	Gene function	References
QTL_1	1	2	LDMP	F, I	34501809–34625326	LOC_Os01g59660	GAMYB (Gibberellin myb gene), leaf senescence	[67]
QTL_2	1	1	FW, TW	I	37942492–38042492		* *	
QTL_3	1	1	FW, TW	I	41033722–41158887	* *		
QTL_4	2	3	FW, TW	F	5726548–6055156	LOC_Os02g10900	RLS1 (rapid leaf senescence 1), chloroplast degradation during leaf senescence	[63]
QTL_5	2	3	FW, TW	F	6162441–6465539			
QTL_6	2	3	FW, TW	I	24075340–24178723	* *		
QTL_7	3	2	FW, TW	I	34570912–34673729	LOC_Os03g60910	OspTAC2, regulation of chloroplast development	[64]
QTL_8	4	5	FW, TW	F	16884687–16935140			
QTL_9	6	3	RTW	F, I	3437953–3786406	* *		
QTL_10	6	3	FW, TW	F, I	17773531–17849604	LOC_Os06g07210	V3 (Virescent3), chloroplast biogenesis	[65]
QTL_11	10	17	FW, TW, DW	F	17189086–18064123	LOC_Os10g32980	OsCESA7 (cellulose synthase A catalytic subunit 7), cellulose synthase	
						LOC_Os10g33310	OsiICK6 (inhibitor of cyclin-dependent kinase 6), involved in cell proliferation to maintain an even growth along the dorsal-ventral plane of leaf blades	[68]
						LOC_Os10g33780	TAW1 (TAWAWA1), controls spikelet number and rice grain yield	[70]
						LOC_Os10g33810	OsMYB110/OsMYB8 (myb transcription factor 8), involved in leaf development and response to abiotic stresses	[69, 83]
QTL_12	12	5	FW, TW	F	6897395–7160358	* *		
QTL_13	12	1	FW, TW	F	26722855–26822855	LOC_Os12g43130	OsPSY2 (Phytoene synthase 2), carotenoid biosynthetic genes	[66]

F: full panel; I: indica subpopulation; FW: leaf fresh weight; TW: leaf turgid weight; DW: leaf dry weight; RTW: relative tissue weight; LDMP: leaf dry matter percentage.

**Fig 3:**
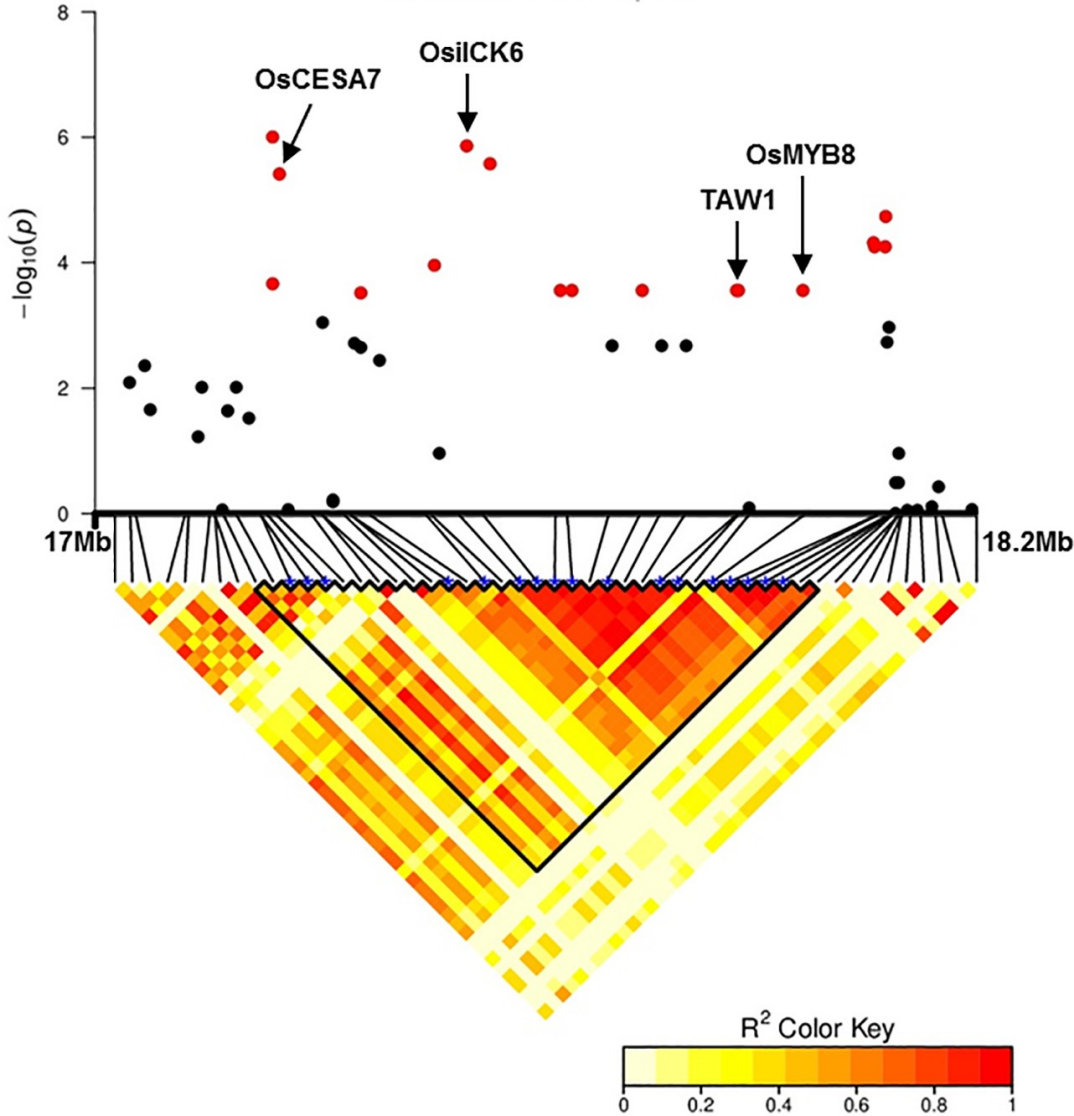
Genomic region of QTL_11 for leaf fresh weight in the full panel, shown in a Manhattan plot and linkage disequilibrium (LD) heat map. In the Manhattan plot, significant SNPs are highlighted in red, and candidate genes of interest are also illustrated. The genomic region of QTL_11 is specified in the boundary area in the LD heat map.

A total of 11 QTLs were identified for FW, 11 for TW, 1 for DW, 1 for RTW and 1 for LDMP. Thus, among the 13 total QTLs, 11 were associated with two or three traits, whereas QTL_1 and QTL_9 were detected for LDMP and RTW, respectively. All 11 of these QTLs were related to both FW and TW (Table 3). Even though strong correlations between FW, TW and DW were observed, only one QTL associated with FW and TW colocalized with DW (i.e., QTL_11). The number of significant SNPs mapped in each QTL mostly varied from 1 to 5, except for QTL_11 on chromosome 10, which was defined by 17 significant markers that separately contributed 7.1% to 13.4% of the phenotypic variation in FW, 7.1% to 13.7% of the phenotypic variation in TW, and 7.3% to 9.1% of the phenotypic variation in DW (Table 3, Table B in S1 Table). Moreover, QTL_11 was also the QTL with the highest P-value peak (P < 10^−6^) in the Manhattan plots (Fig 2A, 2B and 2C). It was located in a large LD block of 875,037 bp (Fig 3).

### Comparison of the QTLs identified in this study with those from previous reports

To identify colocations of QTLs identified in this study with previously published QTLs related to morphophysiological traits derived from mapping populations, we compared physical positions between QTLs using the QTL data from the rice module of TropGeneDB (http://tropgenedb.cirad.fr/tropgene/JSP/interface.jsp?module=RICE). Among the 13 QTLs detected in our study, 11 were found to have overlap(s) with QTLs detected in other studies, whose detailed information is reported in Table C in S1 Table. A total of 92 overlaps were found that belonged to 20 different studies [43–62]. The remaining two QTLs (QTL_4 and QTL_5) did not match known QTLs and thus constitute new QTLs. When comparing the significant associations identified in this study and previous GWAS reports for root-, panicle- and drought-related traits [38–40] using the same rice panel and genotypic data, 37 colocations were also detected (Table D in S1 Table).

### Analysis of candidate genes

Among the 13 identified QTLs, 6 were associated with genes annotated as being related to leaf features (i.e., QTL_1, QTL_4, QTL_7, QTL_10, QTL_11, and QTL_13) (Table 3). In addition to QTL_11, there are two other major QTLs of interest, which are QTL_5 and QTL_12. Nevertheless, according to the functional annotation of the genes within these loci, no candidate genes with an annotated function related to leaf traits could be identified. In general, most of the detected candidate genes such as *RAPID LEAF SENESCENCE 1* (*Os02g10900*) in QTL_4 [63], *PLASTID TRANSCRIPTIONALLY ACTIVE CHROMOSOME PROTEIN 2* (*Os03g60910*) in QTL_7 [64], *VIRESCENT3* (*Os06g07210*) in QTL_10 [65], and *PHYTOENE SYNTHASE 2* (*Os12g43130*) in QTL_13 [66] were predicted to function in leaf physiology. In QTL_1, which was related to LDMP, we found a gene controlling leaf senescence, namely, *GIBBERELLIN MYB GENE* (*Os01g59660*) [67]. In the region with the most significant QTL (QTL_11), three genes reported to be associated with leaf development and biomass production, i.e., *INHIBITOR OF CYCLIN-DEPENDENT KINASE 6* (*Os10g33310*) [68], *MYB TRANSCRIPTION FACTOR 8* (*Os10g33810*) [69], and *CELLULOSE SYNTHASE A CATALYTIC SUBUNIT 7* (*Os10g32980*), as well as a gene (*TAWAWA1*, *Os10g33780*) controlling spikelet number and grain yield [70] were identified.

## Discussion

We phenotyped five leaf-related traits in a panel of 180 Vietnamese rice landraces. High diversity and heritability were observed for four of the investigated traits, with the exception of RTW, for which the variety effect was not significant. The relative constancy of RTW among accessions of a given species might explain this anomaly [42]. Moreover, RTW is linearly related to relative water content [42], the heritability of which was also low, as reported in [40]. In the full panel, strong correlations were observed among the three primary traits (i.e., FW, TW, and DW), which were weakly negatively correlated with LDMP but were not correlated with RTW. Similar phenotypic correlations were observed in the indica and japonica subpopulations. Nevertheless, the japonica subpopulation showed higher mean values than the indica subpopulation for the three primary leaf mass traits, but the values were lower for LDMP and invariable for RTW. This result can be explained by the differences between japonica and indica cultivars in leaf shape and size. As previously reported [71, 72], japonica cultivars have larger leaves but smaller specific leaf areas than indica cultivars.

In this study, we found QTLs for all the investigated traits. QTLs were identified in the full panel and in the indica subpopulation, but no QTLs were detected in the japonica subpopulation, which may be explained by the small size of the japonica subpopulation. This finding was similar to the results reported by [40] for drought related-traits, which used the same panel of Vietnamese rice landraces. There were no common QTLs shared between primary and secondary leaf mass traits (Table 3). Secondary traits are computed from primary traits but reflect specific physiological properties of the plants. Among the secondary traits, RTW showed a very low heritability; consequently, only a single GWAS site was found for this trait. Among the total of 13 QTLs identified in this study, 11 with colocations for FW and TW were observed, while QTL_11 was also related to DW. This result suggests that FW, TW and DW share common genetic determinants. In fact, FW, TW and DW all are parameters of growth, whereas FW expresses biomass accumulation and is directly associated with water content, which reflects the water status of the plant [73], TW is related to water storage capacity, and DW is an indicator of dry matter production and relative growth rate [74].

Our results are consistent with findings of other studies in that overlaps with QTLs related to morphological and physiological characteristics detected in biparental populations were observed. Eleven QTLs of the 13 identified in our study shared similar genomic locations with those of previous studies (Table C in S1 Table). Of the 92 total colocation overlaps, 10 were related to 6 QTLs for FW, TW and DW detected in our study for either a similar trait (leaf fresh weight) [43] or photosynthetic/physiological features of leaves, such as stay-green (i.e., relative retention of leaf greenness, retention of green leaf area, retention degree of leaf greenness, and number of late-discoloring leaves per plant) [56, 57] and transpiration (transpiration rate and carbon isotope discrimination) [49, 55]. The stay-green trait refers to delayed leaf senescence, which reflects the delay of chlorophyll metabolism [75]. A relationship between leaf mass and leaf senescence has been previously reported [76, 77]. Leaf mass and water content significantly decrease during leaf senescence. Similarly, the changes in water lost from leaves due to variation in transpiration efficiency result in changes in leaf mass (i.e., leaf fresh weight) [78]. As such, there is a strong relationship between FW and the transpiration rate. Additionally, transpiration efficiency is largely determined by carbon isotope discrimination [79, 80].

Interestingly, up to 21 colocations of six QTLs identified in this study were associated with yield and yield-related traits (panicle number, spikelet sterility, relative rate of fertile panicles, grain yield, and harvest index) [44, 53, 61]. An explanation might be that the leaf is the main photosynthetic organ accumulating assimilates, which are translocated to the panicle after heading and thus affect grain yield. This confirms that leaf traits can be important variables for yield potential.

In addition, a large number of colocations with QTLs from mapping populations were observed for parameters of plant architecture and growth (e.g., plant height, tiller number, and biomass) [44, 46, 50, 51, 53, 54, 58, 60]. These findings are not surprising because biomass variation in rice plants is mainly dominated by plant height and tiller number, according to the linear regression model reported by [81]. Actually, plant biomass includes leaf biomass. In Arabidopsis, leaf biomass constitutes up to 88% of plant biomass at the vegetative stage; thus, a positive correlation between leaf and plant biomass is observed [82].

As discussed previously, FW and TW are variables of leaf water content and are estimators of plant water status. It is therefore unsurprising that we found many overlaps between our QTLs for these two traits and QTLs for drought stress-related traits from other studies (leaf-rolling score, leaf-drying score, leaf relative water content, osmotic adjustment, proportional water loss required to reach a given leaf-rolling score, relative growth rate, and delay in flowering time under drought stress) [44, 46, 47, 48, 52, 55, 59, 61, 62]. These results suggest that the genetic mechanisms underlying leaf mass parameters are associated with various genetic determinants involved in photosynthesis and transpiration activities, plant growth, yield performance, and stress responses.

Colocations with the associations identified in previous studies using the same Vietnamese rice panel and genotypic data [38–40] were observed (Table D in S1 Table). Of the 454 total associations, 37 associations overlapped with those from all three previous studies. In particular, associations for FW and TW colocated with a rooting depth QTL reported in [38], which is expected, or with a relative crop growth rate QTL from [40]. However, in comparison with results from [39], we found that four QTLs identified for panicle primary branch number colocated on chromosomes 2 and 10 with a number of QTLs we identified for primary leaf mass traits. This finding strengthens the suggestion that leaf parameters can be associated with panicle architecture, which is also a major component of yield.

Underlying the identified QTLs, genes involved in the control of leaf development and biomass production were found within QTL_11 (Fig 3). First, *Os10g33310* (*OsiICK6*) is an inhibitor of cyclin-dependent kinase 6 [68]. The *OsiICK6* gene is expressed in different tissues, including leaves, stems, roots, young panicles and maturing florets, and highly expressed in leaves. *OsilCK6* was reported to be involved in cell proliferation to maintain even growth along the adaxial and abaxial leaf blade surfaces [68]. In contrast to the leaves of control plants, which normally roll slightly toward the adaxial side, the leaves of *OsilCK6*-overexpressing plants roll toward the abaxial side. Second, *Os10g33810 (OsMYB110/OsMYB8)*, which encodes an MYB transcription factor, is involved in leaf development [69] and response to abiotic stresses in rice. As reported by [83], expression of *OsMYB8* in shoots at the seedling stage was strongly induced by cold treatment and lessened by desiccation and wounding. Also underlying QTL_11, *Os10g32980 (OsCESA7)* is a member of the cellulose synthase-like gene superfamily (CESA/CSL) and is proposed to encode an enzyme for cellulose and noncellulosic matrix polysaccharide synthesis in plants. The cellulose synthase complex for cellulose synthesis contains at least three different cellulose synthases encoded by CESA genes [84]. Mutations in any of these genes in rice may cause a significant reduction in the cellulose content of the secondary cell wall, leading to a brittle-culm phenotype [85, 86, 87]. For this reason, Os*CESA7* may be essential for leaf biomass production. In addition to these three genes, in the region of QTL_11, we also found the *Os10g33780 (OsTAWAWA1*) gene, which regulates panicle architecture and development [70]. Its function in the regulation of leaf development should de further studied.

Other genes in the QTLs detected in our study are related to mechanisms of photosynthetic regulation, such as leaf senescence, biogenesis and development of chloroplasts, and carotenoid biosynthesis (Table 3). In plants, photosynthesis occurs in chloroplasts, which contain chlorophyll (green pigment) within thylakoid membranes. Chlorophyll content per unit leaf mass affects the rate of photosynthesis.

In rice, leaf senescence is a physiological phenomenon of programmed cell death happening in the last stage of leaf development [88]. Cell death during leaf senescence is characterized by chloroplast degradation [89]. Regarding this event, *Os02g10900* (*RAPID LEAF SENESCENCE 1 –RLS1*), a nucleotide-binding site-containing protein with an ARM domain, is located within QTL_4 and was reported to be involved in chloroplast degradation during leaf senescence [63]. *Os01g59660* (*GAMYB*), located in QTL_1, encodes an MYB family transcription factor, whose expression level is associated with leaf senescence in rice [67].

Three other candidate genes were found to regulate the generation and development of chloroplasts. *Os03g60910* (*OspTAC2*) is located within QTL_7 and encodes a protein containing 16 PPR repeat domains and an SMR C-terminal domain, which is localized in the chloroplast [90, 91]. *OspTAC2* is highly expressed in young leaves and plays an essential role in normal chloroplast development [64]. The *osptac2* mutant is defective in thylakoid membrane formation, which leads to impaired chloroplast development. *Os06g07210* (*Virescent3* –*V3*) has a function similar to that of *OspTAC2* and encodes the large subunit of ribonucleotide reductase RNRL1, which regulates the rate of deoxyribonucleotide production in DNA synthesis and repair processes. The RNRL1 enzyme was highly expressed in the stem base and young leaves but was altered in *virescent3* (*v3*) mutants [65]. It was indicated that a threshold level of RNRL1 activity is necessary for chloroplast biogenesis during leaf development. In addition, we found within QTL_13 a carotenoid biosynthetic gene, *OsPSY2* (*Os12g43130*), encoding a phytoene synthase 1 that is involved in the terpene synthesis necessary for chloroplast differentiation [66].

The presence of genes involved in the control of leaf development or physiology in some of the detected QTLs helps validate our approach. In this way, our results provide insight into the genetic determinants of leaf mass in rice, which could be used in the future to improve rice yield.

## Supporting information

S1 Table **Table A. List of the 183 rice accessions used in the experiment.** TRAD: traditional; IMP: improved; na: no data available; IR: irrigated; MG: mangrove; RL: rainfed lowland; UP: upland; I: indica; J: japonica; Sub-pop: sub-populations, as defined in [37]. **Table B. GWAS associations and significant markers at P < 5e-04 in the full panel, the indica and the japonica subpopulations.** Chr: chromosome; FW: leaf fresh weight; TW: leaf turgid weight; DW: leaf dry weight; RTW: relative tissue weight; LDMP: leaf dry matter percentage. **Table C. Comparison of the QTLs identified in this study with those previously detected in mapping populations listed in TropGeneDB.** FW: leaf fresh weight; TW: leaf turgid weight; DW: leaf dry weight; RTW: relative tissue weight; LDMP: leaf dry matter percentage. **Table D. Colocalizations of the GWAS associations identified in current and previous studies using the same rice panel and genotypic data.**
(XLSX)

S1 Fig Correlations between leaf mass traits in the three populations. FW: leaf fresh weight; TW: leaf turgid weight; DW: leaf dry weight; RTW: relative tissue weight; LDMP: leaf dry matter percentage.
(TIF)

S2 Fig **Manhattan plots (left) and Q-Q plots (right) for genome-wide association study of leaf mass traits in the indica subpopulation.** A: leaf fresh weight, FW; B: leaf turgid weight, TW; C: leaf dry weight, DW; C: relative tissue weight, RTW; D: leaf dry matter percentage, LDMP.
(TIF)
