# Supplementary figures and images for: Genome-wide association mapping of leaf mass traits in a Vietnamese rice landrace panel
Source: PLoS One. 2019 Jul 8;14(7):e0219274. doi: 10.1371/journal.pone.0219274 (PMC6613685; doi:10.1371/journal.pone.0219274)

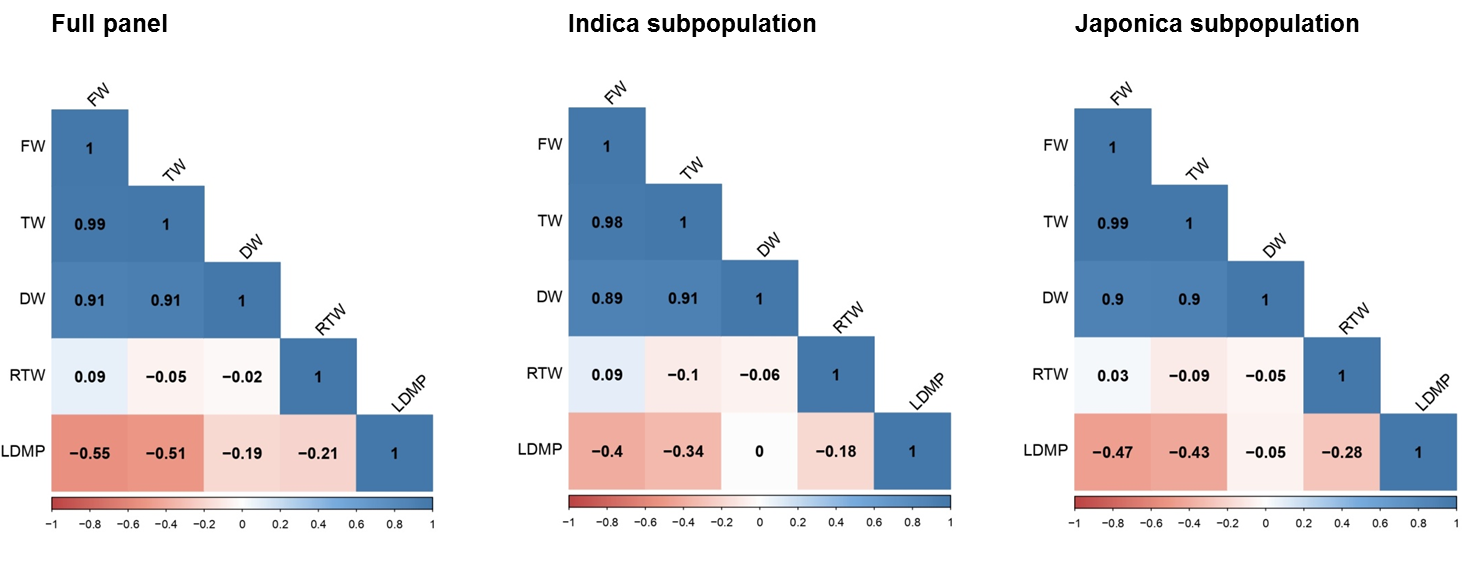

Supplement: S1 Fig — FW: leaf fresh weight; TW: leaf turgid weight; DW: leaf dry weight; RTW: relative tissue weight; LDMP: leaf dry matter percentage. (TIF) [file pone.0219274.s002.tif]

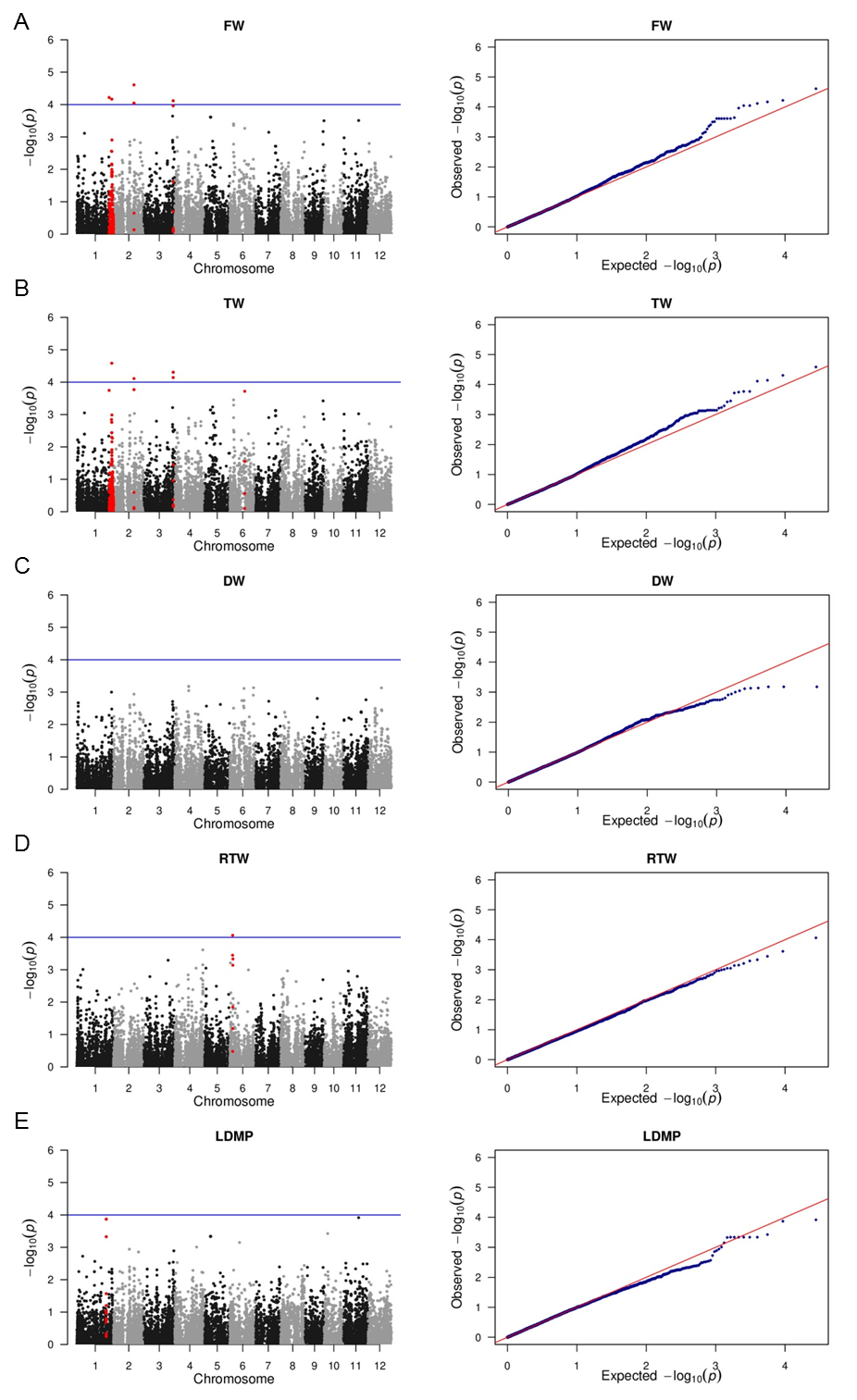

Supplement: S2 Fig — Manhattan plots (left) and Q-Q plots (right) for genome-wide association study of leaf mass traits in the indica subpopulation. A: leaf fresh weight, FW; B: leaf turgid weight, TW; C: leaf dry weight, DW; C: relative tissue weight, RTW; D: leaf dry matter percentage, LDMP. (TIF) [file pone.0219274.s003.tif]
